# Disulfidptosis as an immunometabolic rheostat in gastrointestinal cancers: tuning the balance between T Cell exhaustion and immunogenic cell death

**DOI:** 10.3389/fcell.2026.1932382

**Published:** 2026-08-27

**Authors:** Yachao Li, Huaijue Qiu, Zexing Li, Xiang Gao

**Affiliations:** 1 Department of Gastrointestinal Surgery, Sichuan Provincial People’s Hospital, School of Medicine, University of Electronic Science and Technology of China, Chengdu, Sichuan, China; 2 University of Electronic Science and Technology of China, School of Medicine, Chengdu, China; 3 Department of Emergency, The People’s Hospital of Leshan, Leshan, China; 4 Department of Gastrointestinal Surgery, Sichuan Academy of Medical Sciences & Sichuan Provincial People’s Hospital, University of Electronic Science and Technology of China, Chengdu, China

**Keywords:** dendritic cell cross-presentation, disulfidptosis, gastrointestinal cancers, immunometabolism, immunotherapy, innate immune checkpoint, t cell exhaustion, tumor immune microenvironment

## Abstract

Disulfidptosis is a recently characterized regulated cell death pathway driven by disulfide stress. However, its immunological consequences in the tumor microenvironment remain poorly defined. In this review, we propose a conceptual framework in which disulfidptosis functions as an immunometabolic rheostat, wherein the net outcome—T cell exhaustion versus immunogenic cell death—is critically dependent on stress intensity, kinetics, and cellular context. We hypothesize that in glucose-deprived gastrointestinal tumors, chronic sub-lethal disulfide stress may erode CD8^+^ T cell effector function through F-actin crosslinking at the immunological synapse, potentially involving STAT3–LDHB–G6PD-driven transcriptional reprogramming toward a TOX-associated exhaustion state. Conversely, acute synchronous tumor lysis releases damage-associated molecular patterns (DAMPs); however, productive dendritic cell (DC) cross-presentation requires that adenosine triphosphate (ATP)/adenosine conversion and high mobility group box 1 (HMGB1) redox state meet quantitative thresholds. The DPP7–GPX4 axis suppresses disulfidptosis to limit DAMP release and facilitate natural killer (NK) cell evasion, positioning it as a candidate innate immune checkpoint. We advance testable predictions for tuning this rheostat via timed nanodelivery, dietary sensitization, and DPP7/GPX4 targeting, while explicitly distinguishing correlative biomarkers from causal mechanisms. Priority experiments to validate—or falsify—the rheostat hypothesis are outlined.

## 1 Introduction

### 1.1 The immunotherapeutic challenge in GI cancers and the immune-metabolic barrier

Immune checkpoint blockade (ICB) has revolutionized cancer treatment, yet its efficacy in gastrointestinal (GI) malignancies remains disappointingly limited. Across gastric, colorectal, hepatocellular, pancreatic, esophageal, and biliary tract cancers, objective response rates to anti-PD-1/PD-L1 monotherapy seldom exceed 15%–20%; in pancreatic cancer, they languish below 5% (Llovet et al., 2022; Le et al., 2017). This poor performance stems from a fundamental immunological feature: most GI tumors present a “cold” tumor immune microenvironment (TIME), characterized by sparse CD8^**+**^ T cell infiltration, abundant immunosuppressive myeloid cells, and a dense desmoplastic stroma that physically and metabolically excludes effector lymphocytes (Wherry and Kurachi, 2015).

The failure of ICB in GI cancers is not merely a matter of insufficient T cell numbers, but a failure of T cell functional quality. Unlike hematological malignancies or melanomas—which often exhibit high mutational burden, neoantigen load, and pre-existing T cell infiltration with preserved effector function—GI cancers undergo substantial metabolic reprogramming. The Warburg effect drives avid glucose consumption and lactate production, resulting in extracellular acidification and severe nutrient competition. Such metabolic rewiring directly impairs effector T cell metabolism: under glucose-limited conditions, T cells cannot sustain oxidative phosphorylation and instead rely on glycolysis, which is insufficient to meet the energetic demands of persistent cytokine production and cytotoxicity. Consequently, intratumoral CD8^+^ T cells undergo progressive terminal exhaustion. This state is marked not only by sustained co-expression of programmed death-1 (PD-1), T cell immunoglobulin and mucin domain-3 (TIM-3), lymphocyte activation gene-3 (LAG-3), and the transcription factor TOX, but more critically by progressive loss of T cell receptor (TCR) signaling capacity, impaired immunological synapse stability, diminished cytolytic granule release, and erosion of memory precursor potential (Wherry and Kurachi, 2015). This metabolic barrier represents a primary hurdle for ICB efficacy.

### 1.2 Disulfidptosis: aproposed immunometabolic rheostat at the core of T cell dysfunction and tumor immunogenicity

The central hypothesis of this review is that disulfidptosis may serve as a metabolic fulcrum with dual potential: it is not merely a tumor cell death subroutine, but a pathway that may determine whether infiltrating lymphocytes are empowered or silenced (Liu et al., 2023; Liu et al., 2024; Xiao et al., 2024). On one side, chronic sub-lethal disulfide stress is hypothesized to be associated with progressive erosion of CD8^+^ T cell function, impairing TCR signaling, destabilizing the immunological synapse, and correlating with TOX-mediated epigenetic remodeling. On the other side, acute synchronous disulfidptosis in tumor cells can release DAMPs that, if quantitatively sufficient, may prime DC maturation and cross-presentation. The molecular engine behind both outcomes is the same: the SLC7A11/NADPH redox axis. Under glucose deprivation, nicotinamide adenine dinucleotide phosphate (NADPH) depletion triggers aberrant disulfide crosslinking of actin-binding proteins (filamin A [FLNA], filamin B [FLNB], drebrin), collapsing the F-actin cytoskeleton (Liu et al., 2023). However, we propose that the immunological consequence is not determined by this molecular event alone, but by the cellular context, intensity, and kinetics of the stress—a concept we develop here as the “immunometabolic rheostat”.

#### 1.2.1 Conceptual framework of the rheostat

**FIGURE 1:**
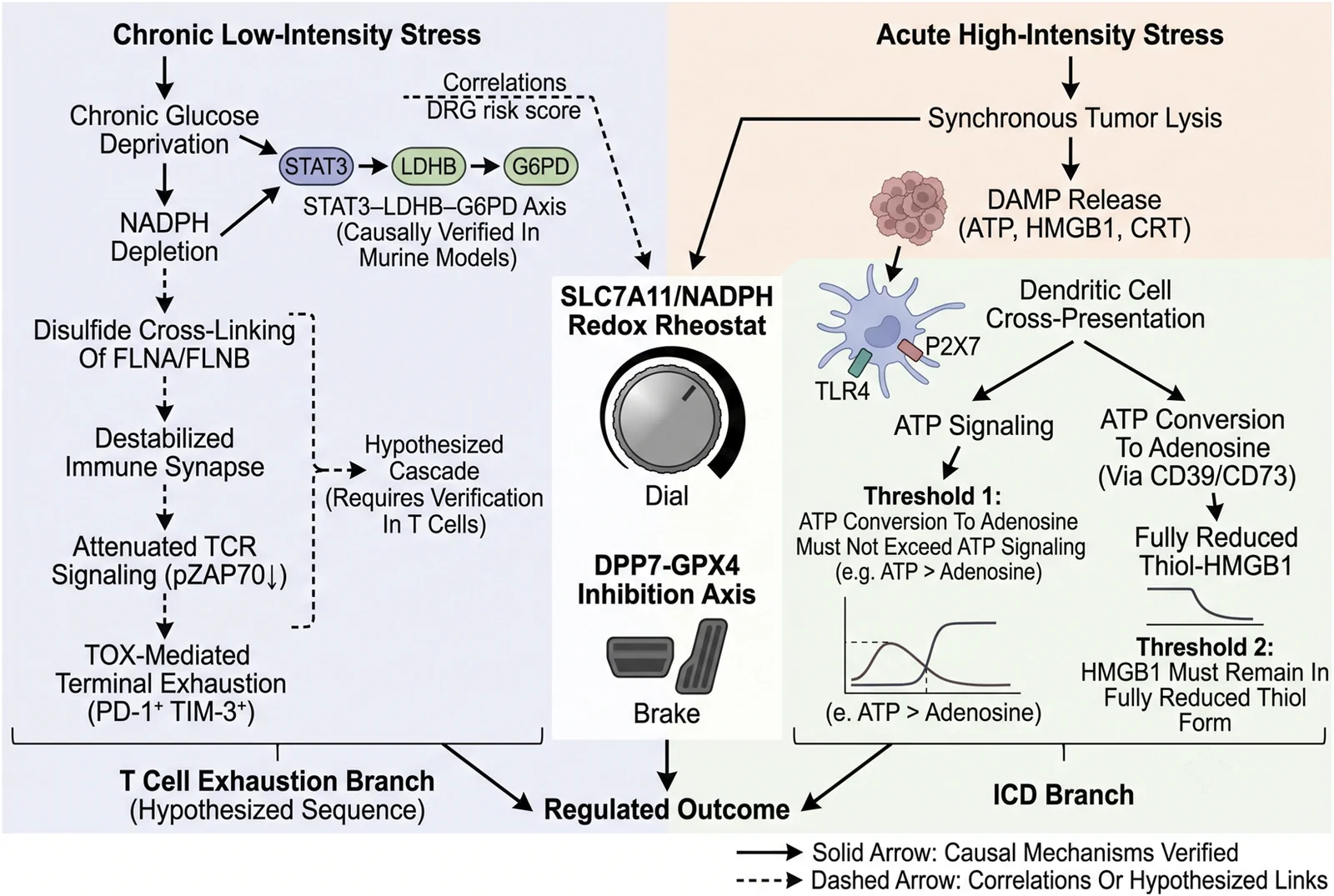
Disulfidptosis as an immunometabolic rheostat. The schematic illustrates the proposed threshold-based model. Chronic glucose deprivation (left) depletes NADPH, promotes disulfide crosslinking of actin-binding proteins (FLNA/FLNB), destabilizes the immunological synapse, reduces TCR signalling (pZAP70↓), and drives TOX-mediated terminal exhaustion (PD-1^+^ TIM-3^+^). Acute high-intensity stress (right) induces synchronous tumour lysis and releases DAMPs (ATP, HMGB1, CRT). For dendritic cells to cross-present via P2X7 and TLR4, two thresholds must be met: ATP breakdown to adenosine (via CD39/CD73) must not overwhelm ATP signalling, and HMGB1 must remain in its fully reduced thiol form. The SLC7A11/NADPH redox rheostat and the DPP7–GPX4 inhibitory axis modulate the net outcome. Solid arrows denote causally validated mechanisms (e.g., STAT3–LDHB–G6PD in murine T cells); dashed arrows indicate correlative or hypothetical links (e.g., DRG risk scores).

To guide this integration, we propose a threshold-based model (see Graphical Abstract). At low-intensity, chronic stress (e.g., persistent glucose scarcity in the tumor core), disulfide accumulation in CD8^**+**^ T cells is hypothesized to cause partial F-actin crosslinking—sufficient to destabilize the immunological synapse and impair TCR signaling, but not to induce immediate cell death. This drives a progressive, TOX-associated transcriptional exhaustion program. At high-intensity, acute stress (e.g., following pharmacologically synchronized metabolic crisis), tumor cells undergo rapid lytic death, releasing DAMPs (ATP, HMGB1) en masse. Whether this tips the balance toward immunogenic cell death depends on two downstream kinetic thresholds: (i) the ATP/adenosine conversion rate, and (ii) the redox state of HMGB1. This framework reframes the central question from “Is disulfidptosis immunogenic?” to “Under what precise spatiotemporal conditions does it become so?” (Li and Gan, 2025) (Figure 1).

### 1.3 Scope and rationale of this review

Existing literature on disulfidptosis has largely focused on its molecular execution—actin cytoskeletal collapse, NADPH depletion, and SLC7A11 dependency—with cancer biology reviews emphasizing tumor-cell-autonomous effects (Liu et al., 2024; Li et al., 2024). However, a systematic immunological synthesis remains absent. No existing framework has reconciled the pathway’s dual capacity to both impair intratumoral lymphocyte function and provoke tumor immunogenicity, nor has the field articulated testable conditions under which one outcome predominates over the other.

This review fills that gap by advancing the “immunometabolic rheostat” hypothesis. Rather than providing an exhaustive catalog of all published findings, we prioritize immune-centric mechanisms: TCR signaling integrity, DC cross-presentation, innate lymphocyte regulation, and memory differentiation. We explicitly distinguish correlative evidence from causal mechanisms, highlight critical missing links, and propose a prioritized experimental agenda to validate or refute our framework. By synthesizing evidence across GI malignancies—colorectal, gastric, hepatocellular, pancreatic, esophageal, and biliary tract cancers—we aim to identify unifying principles that inform rational immunotherapeutic design (Chen Y. et al., 2025). In doing so, we contextualize our proposed framework within recent comprehensive reviews on disulfidptosis and redox biology, including those addressing pharmacological modulation and systemic metabolic consequences (Chen R. et al., 2025; Zhang et al., 2025; Qian et al., 2026; Qian et al., 2024).

## 2 The metabolic core of disulfidptosis: a primer for immunologists

For immunologists, the relevant essence of the disulfidptosis machinery can be distilled into three interconnected principles.

### 2.1 First, the SLC7A11/NADPH axis is the redox rheostat

SLC7A11 (xCT) imports cystine in exchange for glutamate. In glucose-replete conditions, cystine fuels glutathione (GSH) synthesis, and NADPH regenerates GSH via glutathione reductase, maintaining redox balance. Under glucose deprivation—a hallmark of the poorly vascularized GI TIME—NADPH production via the pentose phosphate pathway plummets, but SLC7A11 continues to drive cystine influx. The resultant imbalance causes aberrant disulfide crosslinking of actin-binding proteins (FLNA, FLNB, drebrin), triggering F-actin collapse (Liu et al., 2023; Qiu et al., 2025). Critically, this collapse has diametrically opposed immunological effects depending on the cell type: in tumor cells, it causes lytic death with potential DAMP release; in T cells, it may disrupt the actin cytoskeleton required for immunological synapse stability and cytotoxic granule polarization, impairing effector function without immediate lysis. This differential susceptibility, as we discuss in Sections 3 and 4, is hypothesized to arise from distinct NADPH compartmentalization and SLC7A11 expression levels between tumor cells and lymphocytes, though direct quantitative comparisons in the same tumor bed are currently lacking (Qiu et al., 2025).

### 2.2 A critical unresolved question concerns cell-type-specific outcomes

In tumor cells, disulfide stress typically culminates in lytic death. In T cells, the same pathway appears to produce functional paralysis without immediate lysis. We propose three non-mutually exclusive explanations. First, tumor cells often express markedly higher SLC7A11 levels than lymphocytes, driving cystine influx that overwhelms cytoskeletal buffering capacity. Second, NADPH compartmentalization may differ—T cells rely heavily on cytosolic NADPH from the pentose phosphate pathway, which is acutely glucose-sensitive, whereas tumor cells may maintain mitochondrial NADPH pools via isocitrate dehydrogenase 2 (IDH2) and malic enzyme under glucose deprivation (though this remains unquantified in the GI TIME). Third, actomyosin dynamics differ fundamentally: a sessile tumor cell and a motile T cell face vastly different consequences from the same absolute level of F-actin crosslinking. These remain hypotheses, not established facts, and we explicitly call for direct comparative measurements in Section 5.

### 2.3 Second, DPP7–GPX4 constitutes a tumor-intrinsic brake on disulfidptosis that may directly enable innate immune evasion

Dipeptidyl peptidase 7 (DPP7) physically stabilizes glutathione peroxidase 4 (GPX4) protein (independent of transcriptional regulation), blunting disulfide stress and preventing actin crosslinking. This suppression of disulfidptosis reduces DAMP release and renders tumor cells less susceptible to NK-cell-mediated killing in preclinical models (Li R. et al., 2025). We return to this axis in Section 4.3 as a candidate innate immune checkpoint, while acknowledging that its role as a true checkpoint—analogous to PD-L1—requires further validation.

### 2.4 Third, the pathway is pharmacologically and nutritionally malleable

Small-molecule sensitizers (e.g., lomerizine, clioquinol) and desensitizers (e.g., nicotinamide riboside chloride [NR-CL]) can modulate the NADPH/GSH balance, offering avenues to precisely tune disulfidptosis intensity—a feature that distinguishes it from genetically fixed death pathways (Chen R. et al., 2025; Zhang et al., 2025). We refer readers to comprehensive mechanistic reviews for detailed detection methodologies; here, we focus on immunological implications.

## 3 A proposed role for disulfidptosis in T Cell exhaustion: evidence and open questions

### 3.1 DRG signatures as readouts of T cell fitness and immune exclusion

Across GI malignancies, the immunological state of the TIME—particularly the functional competence of tumor-infiltrating CD8^**+**^ T cells—is consistently reflected in transcriptional signatures derived from disulfidptosis regulators (DRGs; hereafter referred to as DRG scores). In colorectal, gastric, hepatocellular, and pancreatic cancers, risk scores based on DRGs (e.g., RAB7A, SLC7A11, INF2, FLNA) stratify patients into groups with profoundly distinct immune profiles (Zhang et al., 2025; Jiang et al., 2026; Yu et al., 2025; Wang Y. et al., 2025; Xu et al., 2026; Li Q. et al., 2025). In low-risk groups, intratumoral T cells retain effector characteristics: higher CD8^**+**^ density, reduced PD-1/TIM-3 co-expression, preserved TCR signaling capacity (inferred from phospho-STAT1/IRF1 signatures), and enrichment for CD56bright NK cells and γδT cells. This configuration correlates with improved ICB responses. In high-risk groups, the T cell compartment is marked by terminal exhaustion: PD-1^**+**^ CD8^**+**^ T cells co-expressing TOX, enriched M2 macrophages, and myeloid-derived suppressor cell (MDSC) accumulation—a “cold” TIME refractory to checkpoint blockade (Zhang et al., 2025; Jiang et al., 2026; Yu et al., 2025; Wang Y. et al., 2025; Xu et al., 2026; Li Q. et al., 2025).

It is critical to recognize that these signatures are correlative predictors, not causal drivers. The DRG risk score does not cause T cell exhaustion; rather, it integrates the cumulative transcriptional consequence of metabolic stress on infiltrating lymphocytes and stromal cells. Thus, DRG signatures function as surrogate biomarkers of immune fitness.

### 3.2 The STAT3–LDHB–G6PD axis in CD8^+^ T cell dysfunction: a mechanistically plausible but incompletely validated model

While SLC7A11 is traditionally studied in tumor cells, its role in immune compartments is equally critical and mechanistically distinct. In the glucose-deprived GI TIME, infiltrating CD8^+^ T cells upregulate SLC7A11 expression as an adaptive attempt to maintain GSH levels. However, this compensatory response may become maladaptive under persistent glucose scarcity. Excessive cystine influx, coupled with NADPH depletion, is hypothesized to trigger disulfide stress specifically within actin-binding proteins of the T cell cytoskeleton.

At the molecular level, chronic disulfide stress is proposed to drive a progressive decline in effector capacity through three interconnected mechanisms, each supported by varying levels of evidence.

#### 3.2.1 Synaptic disruption

F-actin remodeling is essential for synapse formation. In non-immune cells, disulfide crosslinking of actin-binding proteins (FLNA, FLNB) collapses the F-actin cytoskeleton. By extrapolation—which remains to be tested directly in T cells—this could disrupt the synaptic actin network, potentially impairing TCR–pMHC clustering and the localization of co-stimulatory molecules. Consistent with this hypothesis, tumor-infiltrating lymphocytes from high-DRG-risk patients exhibit reduced pZAP70 levels. However, formal demonstration that this results from F-actin crosslinking *per se*, rather than from other redox-sensitive TCR-proximal events, is lacking. Beyond cytoskeletal disruption, disulfide stress may directly impair TCR-proximal signaling. LCK, a Src-family kinase, has two conserved cysteines (Cys^420^ and Cys^478^) that are essential for catalytic activity. When NADPH/GSH are depleted, these cysteines can be sulfenylated or sulfonylated. This modification reduces LCK activity and dampens downstream phosphorylation of CD3ζ and ZAP70. This suggests a dual-hit scenario—synaptic F-actin collapse plus biochemical inhibition—that could silence TCR signaling at two levels, but definitive evidence in human tumor-infiltrating T cells is still awaited.

#### 3.2.2 Impaired cytotoxic granule polarization

Effective target cell killing requires trafficking of cytolytic granules toward the immunological synapse. Disulfide-mediated F-actin collapse would be expected to prevent granule polarization, reducing the efficiency of target cell lysis. This mechanism has been observed in other settings of cytoskeletal disruption, but direct evidence linking disulfidptosis to granule mistrafficking in CD8^+^ T cells is not yet published.

Transcriptional reprogramming toward exhaustion is directly mediated by the STAT3–LDHB–G6PD signaling cascade, based on compelling murine genetic data. Signal transducer and activator of transcription 3 (STAT3) directs lactate dehydrogenase B (LDHB) expression, which in turn restricts glucose-6-phosphate dehydrogenase (G6PD) activity and NADPH generation, creating a permissive state for disulfidptosis in exhausted CD8^**+**^ T cells. This is associated with the transcriptional upregulation of exhaustion markers (PD-1, TIM-3, TOX) and skews the T-bet/Eomes balance toward T-bet^low^ Eomes^high^ terminal exhaustion. Genetic ablation of Ldhb in T cells prevents disulfidptosis-associated exhaustion and preserves effector cytokine production in murine models (Li and Gan, 2025; Wan et al., 2025; Senft, 2025). This provides proof-of-concept that LDHB is a therapeutically tractable node, though translation to human T cells and GI-specific contexts remains to be established.

#### 3.2.3 Temporal hierarchy among the three mechanisms

We propose, as a testable hypothesis, that these events do not occur simultaneously but follow a stress-intensity-dependent cascade. Under mild glucose deprivation, the earliest event may be transcriptional reprogramming via STAT3–LDHB–G6PD, which primes T cells for a permissive chromatin state (TOX upregulation) without immediately impairing synapse formation. As stress persists, synaptic disruption may emerge as a second-hit amplifier, physically uncoupling TCR engagement from downstream signaling. Only when disulfide stress reaches a lytic threshold might granule polarization failure supervene, abolishing cytotoxic capacity. This hierarchy predicts that early pharmacological interception of STAT3–LDHB may prevent the downstream structural defects, whereas late intervention (after F-actin crosslinking) would be ineffective—a testable prediction outlined in Box 1.

#### 3.2.4 Evidence gaps and causal relationships

Throughout this review, we distinguish between correlative observations derived from bulk transcriptomic risk scores, and causally validated mechanisms established through genetic ablation, adoptive transfer, or pharmacological rescue. Currently, the STAT3–LDHB–G6PD axis has been causally linked to T-cell exhaustion in murine models, with Ldhb deletion in T cells preventing exhaustion, whereas DRG risk scores and DPP7 expression correlations with NK infiltration remain associative. Several hypotheses require direct experimental testing, including whether T-cell-specific Slc7a11 ablation rescues TCR signaling and effector function *in vivo*, and whether disulfidptosis meets the quantitative DAMP thresholds required for *bona fide* immunogenic cell death.

### 3.3 Differential metabolic wiring of tregs and innate-like T cells: testable hypotheses

While CD8^+^ T and NK cells have garnered the most attention, the net immunological outcome of disulfidptosis is likely shaped by additional populations whose metabolic wiring renders them differentially susceptible to disulfide stress. We frame these as testable hypotheses rooted in their distinct metabolic dependencies.

Regulatory T cells (Tregs) present a double-edged puzzle. Unlike effector T cells, Tregs rely more on fatty acid oxidation and resist glucose deprivation—features that may grant them a survival advantage in glucose-poor niches. However, FOXP3 stability demands high NADPH pools to maintain reduced GSH and counteract reactive oxygen species (ROS). In a disulfidptosis-prone environment with limiting NADPH, we propose two competing hypotheses. Tregs may be preferentially depleted, relieving immunosuppression. Alternatively, Tregs may upregulate mitochondrial NADPH production via IDH2 to withstand disulfide stress, leading to relative Treg enrichment (Josefowicz et al., 2012). Distinguishing these requires fate-mapping in Foxp3-reporter mice subjected to pharmacological disulfidptosis induction, coupled with functional suppression assays to assess whether surviving Tregs retain regulatory capacity—experiments that are conspicuously absent from the current literature.

γδT and invariant natural killer T (iNKT) cells are abundant in GI mucosal tissues and respond to metabolic stress and DAMP signals (ATP, HMGB1) via P2X7 and Toll-like receptor 4 (TLR4). Notably, γδT cells are less glucose-dependent than conventional αβT cells, relying instead on mevalonate metabolism and pre-programmed effector functions (Godfrey et al., 2018). This suggests they might resist disulfidptosis-mediated exhaustion and could serve as a functional reservoir in glucose-deprived microenvironments. We propose that the γδT-to-αβT ratio within GI tumors—an emerging correlate of ICB responsiveness—should be examined in relation to DRG scores, as a higher ratio may predict relative preservation of antitumor activity under metabolic stress.

By framing these not as established facts but as experimentally addressable hypotheses, we provide a roadmap for dissecting the full immunological impact of disulfidptosis beyond the CD8^+^ T-cell and NK-cell compartments.

## 4 Dual immunological edges: from tumor lysis to DC/NK activation

### 4.1 Metabolic heterogeneity dictates differential disulfidptosis vulnerability across GI subtypes

The heterogeneous metabolic landscape across GI tumors directly dictates their intrinsic “disulfidptosis vulnerability” and, consequently, their propensity to undergo immunogenic cell death. Pancreatic ductal adenocarcinoma, characterized by extreme stromal fibrosis, hypovascularity, and chronically glucose-deprived niches, exhibits intermediate disulfidptosis activity. Intriguingly, patients in this subgroup show heightened sensitivity to anti-PD-L1 therapy in some correlative analyses, suggesting that pre-existing metabolic stress may prime tumors for disulfidptosis-induced immunogenicity. However, this remains an associative finding requiring prospective validation. In hepatocellular carcinoma, ketogenic diet-induced metabolic adaptation enhances disulfidptosis sensitivity under glucose-starved conditions via NRF1/NRF2 antagonism, offering a dietary sensitization strategy (Zhang et al., 2025). In colorectal cancer, GLUT1 inhibition reverses glucose-driven metastasis and, when combined with anti-PD-1, promotes CD8^+^ T cell recruitment—an effect mechanistically linked to FLNA-mediated cytoskeletal remodeling (Li Q. et al., 2025). Esophageal squamous cell carcinoma, though less studied, has yielded two distinct disulfidptosis expression patterns with divergent overall survival. Thus, rather than a one-size-fits-all model, disulfidptosis sensitivity appears to be a function of each tumor’s intrinsic metabolic wiring—determined by glucose availability, stromal architecture, oncogenic drivers, and genetic background (Chen Y. et al., 2025).

Although the above landscape is qualitatively consistent across studies, direct quantitative comparisons of NADPH/NADP^+^ ratios and SLC7A11 protein levels between GI subtypes remain scarce. Where available, we summarize representative values in Table 1 (rightmost column) to anchor the rheostat threshold in measurable units.

**TABLE 1:** Baseline TIME features, DRG-associated phenotypes, and ICB responsiveness across major GI cancers.

Cancer Type	Baseline TIME Profile	DRG High-Risk Phenotype	DRG Low-Risk Phenotype	Known ICB Response Rate	Key Quantitative Evidence (NADPH/SLC7A11) Relevant to Disulfidptosis
Colorectal (pMMR/MSS)	Cold; low CD8^+^; high TAMs	M2^+^, MDSC^+^, PD-1^+^CD8^+^ exhausted	CD56bright NK^+^, γδT^+^, CTLA-4^+^	<5% (anti-PD-1 monotherapy)	SLC7A11 is frequently upregulated in MSS tumors; NADPH/NADP^+^ balance is skewed toward oxidation in tumor lysates relative to adjacent tissue (Jiang et al., 2026; Li Q. et al., 2025)
Gastric	Mixed (EBV^+^/CIN subtypes)	Enriched in Wnt/ECM pathways	High CD8^+^; low MDSC	∼10–15% (nivolumab monotherapy, unselected)	HER2^+^ subtype shows higher SLC7A11 expression than HER2^-^; intratumoral glucose is markedly lower than plasma levels (Yu et al., 2025; Wang J. et al., 2025)
Hepatocellular	Intermediate; Treg-rich	M2^+^, exhausted CD8^+^	M1-like macrophages; NK^+^	∼14–17% (anti-PD-1 monotherapy)	Ketogenic diet sensitizes tumors by lowering NADPH/NADP^+^; NRF1/2 antagonism reduces SLC7A11 protein expression (Zhang et al., 2025)
Pancreatic	Cold; desmoplastic; MDSC-rich	Low CD8^+^; high cancer-associated fibroblasts	CD8^+^ infiltration (rare)	<5% (anti-PD-1 monotherapy)	SLC7A11 is overexpressed in PDAC compared with normal ducts; stromal glucose is severely depleted in core regions; NADPH pools are substantially lower than in adjacent stroma ( Xu et al., 2026; Li Q. et al., 2025; Wang J. et al., 2025; Senft, 2025; Josefowicz et al., 2012 ; Xu et al., 2026; Li W. et al., 2026; Yin J. et al., 2025; Yin Y. et al., 2025; Shi et al., 2025)
Cholangiocarcinoma	Varied; often immune-excluded	Higher infiltration but worse prognosis (editing)	Not well characterized	∼5–20% (ICI monotherapy, highly variable by subtype)	SLC7A11 high expression is observed in a subset of cases and correlates with worse overall survival (Chen R. et al., 2025)

The rightmost column summarizes qualitative evidence from published studies; direct quantitative comparisons across subtypes are largely absent. ICB, response rates in the fourth column refer to anti-PD-1/PD-L1, monotherapy in unselected patient populations unless otherwise specified. These descriptions are intended to anchor the rheostat concept in measurable biological contexts and should be interpreted as hypothesis-generating.

### 4.2 The dendritic cell cross-presentation bottleneck

For disulfidptosis to elicit productive antitumor immunity, the death of tumor cells must translate into T cell priming—a process that depends entirely on DCs, particularly CD103^+^ conventional DC type 1 (cDC1s). From the DC’s perspective, the immunogenicity of disulfidptosis is not guaranteed by DAMP release alone; it is governed by three quantitative checkpoints that are largely inferred from studies of other cell death pathways, with limited direct data for disulfidptosis.

#### 4.2.1 First, the concentration and kinetics of ATP and HMGB1 must exceed activation thresholds

Rapid, synchronous tumor lysis releases ATP (within minutes) and HMGB1/calreticulin (within hours). ATP at concentrations >100 µM signals through P2X7 on DCs to promote NLRP3 inflammasome activation and interleukin-1β (IL-1β) secretion; HMGB1 in its fully reduced form (all three cysteines in thiol state) binds TLR4 with high affinity to drive MyD88-dependent DC maturation (Xu S. et al., 2025). However, disulfidptosis occurs in a glucose-deprived, highly oxidizing microenvironment that may rapidly convert HMGB1 to its disulfide or sulfonyl isoforms, which are poor TLR4 agonists or even antagonists. Quantifying the reduced-to-oxidized HMGB1 ratio in the tumor interstitial fluid—rather than total HMGB1 levels—will be essential to predict whether disulfidptosis elicits immunogenic cell death or tolerance (Galluzzi et al., 2024; Galluzzi et al., 2020; Li C. et al., 2026).

Second, the ATP-to-adenosine conversion rate, dictated by CD39/CD73 ectonucleotidase activity, determines whether ATP signals as a “find-me” cue or as a tolerogenic brake. In glucose-deprived niches, CD73 is often upregulated via hypoxia-inducible factor-1α (HIF-1α), rapidly degrading ATP to adenosine, which suppresses DC maturation via A2A receptors. Without concurrent ectonucleotidase inhibition, even high ATP release may be functionally neutralized (Liu et al., 2025). The net effect of ATP release during disulfidptosis therefore depends on the ATP-to-adenosine conversion rate—a kinetic parameter shaped by CD39/CD73 expression on tumor cells, stromal fibroblasts, and regulatory immune cells.

#### 4.2.2 Third, DCs themselves may be direct targets of disulfide stress

cDC1s depend on lipid metabolism and redox balance for major histocompatibility complex (MHC) class I recycling and migratory capacity. Whether NADPH depletion impairs cDC1 survival or cross-presentation efficiency in the disulfidptosis milieu is unknown. Thus, we propose that the immunological outcome of disulfidptosis is not determined by tumor cell death *per se*, but by whether the DAMP signals reach DCs in sufficient concentration, in the right redox form, and before being degraded—and whether DCs retain functional competence to process and present antigens (Galluzzi et al., 2024; Galluzzi et al., 2020; Li C. et al., 2026).

#### 4.2.3 Recent direct evidence linking disulfidptosis to DAMP-mediated immunogenic cell death

A landmark study by Huang et al. (2026) demonstrated that RRx-001, a clinical-stage small-molecule agent, markedly reduces NADPH levels by downregulating G6PD, leading to redox imbalance and F-actin cytoskeletal contraction—hallmarks of disulfidptosis. Critically, RRx-001 promoted the release of DAMPs, including calreticulin (CRT), HMGB1, and HSP70/90, thereby activating immunogenic cell death (ICD). *In vivo*, RRx-001 significantly suppressed tumor growth, enhanced infiltration of CD4^**+**^ and CD8^**+**^ T cells, increased M1 macrophage polarization, and downregulated PD-L1 expression in hepatocellular carcinoma models (Huang et al., 2026). This study provides the first direct evidence that pharmacological induction of disulfidptosis can elicit *bona fide* DAMP-mediated ICD and productive antitumor immunity, and serves as a critical reference for future comparative studies. Nevertheless, it is important to note that according to consensus guidelines for immunogenic cell death, rigorous demonstration of *bona fide* ICD requires not only DAMP release and *in vivo* tumor growth inhibition, but also functional vaccination assays, tumor rechallenge experiments, and evidence of antigen-specific T-cell memory (Galluzzi et al., 2020). These criteria have not yet been fully met for disulfidptosis in most GI models, and we explicitly highlight this as a priority for future investigation.

### 4.3 The DPP7–GPX4 axis as a candidate innate immune checkpoint: NK cell evasion via disulfidptosis suppression

**FIGURE 2:**
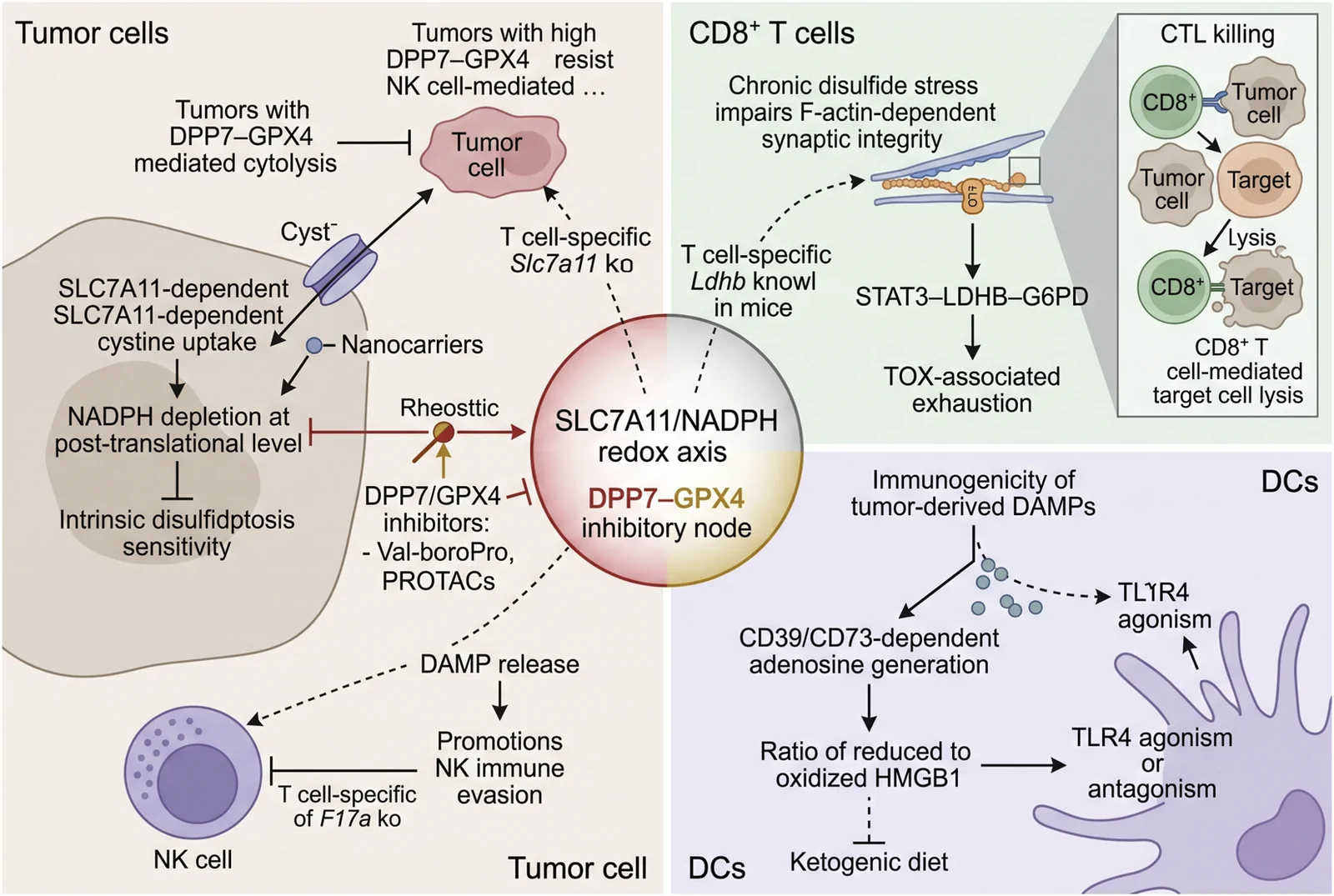
Intercellular and molecular network of disulfidptosis in the gastrointestinal TIME. The schematic links four compartments—tumour cells, CD8^+^ T cells, DCs and NK cells—within the glucose-deprived gastrointestinal microenvironment. In tumour cells (left), SLC7A11-dependent cystine uptake and NADPH depletion govern intrinsic disulfidptosis sensitivity, whereas the DPP7–GPX4 axis suppresses the pathway post-translationally, curtailing DAMP release and facilitating NK evasion. In CD8^+^ T cells (upper right), chronic disulfide stress impairs F-actin-dependent synapse integrity and activates STAT3–LDHB–G6PD signalling, promoting TOX-associated exhaustion. For DCs (lower right), the immunogenicity of tumour-derived DAMPs is gated by CD39/CD73-dependent adenosine production and the reduced/oxidized HMGB1 ratio, which determine TLR4 agonism versus antagonism. DPP7–GPX4-high tumours resist NK cytolysis. Solid lines indicate experimentally established pathways (e.g., Ldhb ablation in mice); dashed lines denote hypotheses requiring direct human validation, including T-cell-specific Slc7a11 ablation and spatial correlation of FLNA aggregates with pZAP70^+^ infiltrates. Pharmacological nodes—nanocarriers, DPP7/GPX4 inhibitors (Val-boroPro, PROTACs) and ketogenic diets—are points for rheostat tuning. All figures were originally designed and drafted by the authors; no AI-generated artwork was used. In both figures, solid arrows represent experimentally validated pathways (e.g., genetic ablation studies in murine models), whereas dashed arrows denote correlative associations or hypotheses that await direct experimental confirmation in human GI tissues or in vivo T-cell-specific models.

Natural killer (NK) cells are poised to eliminate tumor cells that downregulate MHC class I or upregulate stress ligands. However, GI tumors frequently evade NK surveillance through a mechanism that is conceptually analogous to PD-L1 in the adaptive compartment: the DPP7–GPX4 axis. In colorectal, gastric, and hepatocellular carcinomas, high DPP7 expression correlates with poor prognosis and reduced intratumoral CD56^**+**^ cell density. DPP7 physically interacts with GPX4 to stabilize it post-translationally (without altering mRNA levels). This stabilization maintains redox balance and blunts disulfide stress, preventing F-actin collapse and lytic death. Functionally, DPP7-high tumor cells release fewer DAMPs and are markedly less susceptible to NK-cell-mediated cytolysis; GPX4 restoration in DPP7-deficient cells fully reverses this sensitization. Thus, by suppressing disulfidptosis, the DPP7–GPX4 axis may eliminate the DAMP signals that would otherwise recruit NK cells and activate their cytotoxic program (Li R. et al., 2025). We therefore propose DPP7 and GPX4 as co-targets for a novel immunotherapeutic strategy that breaks innate immune evasion and unlocks subsequent adaptive responses. However, it is important to note that DPP7–GPX4 functions primarily as a tumor-intrinsic redox-survival mechanism; its designation as an “innate immune checkpoint” is a conceptual proposal that requires formal validation through functional studies demonstrating that its blockade enhances NK-cell-mediated tumor clearance in an immune-dependent manner. Furthermore, GPX4 inhibition carries on-target toxicity to intestinal epithelium, necessitating tumor-selective delivery. Most of the evidence for this axis derives from a limited number of preclinical models; broader validation across human GI subtypes is still needed (Figure 2).

## 5 Therapeutic strategies, bottlenecks, and resistance

### 5.1 Nano-inducers, dietary interventions, and rational combination with ICB

Advances in nanodelivery have provided precise tools to exploit the metabolic vulnerability of disulfidptosis. Disulfidptosis nano-inducers (e.g., CYBC nanoparticles) act through two coordinated mechanisms: they block glucose uptake via GLUT1 inhibition and supplement extracellular cystine. This dual action triggers disulfide stress, collapses the cytoskeleton, and redirects tumor metabolism toward immunogenic cell death (Xu Y. et al., 2025). Copper-based platforms like CuSS@876-PEG and CST nanoparticles exploit GSH depletion, copper ion release, and glucose transporter inhibition to co-activate cuproptosis and disulfidptosis (Jin et al., 2025). Transformable albumin nanocapsules and intermetallic nanoreactors (e.g., Pd_2_Sn@GOx-SP and ITG@PCM) have similarly been engineered to co-induce disulfidptosis with pyroptosis, releasing a broad spectrum of DAMPs. Oxygen-driven Prussian blue nanomotors have also been developed to disrupt redox state and induce disulfidptosis for tumor immunotherapy (Yang et al., 2025). Additionally, inhibition of endoplasmic reticulum stress cooperates with SLC7A11 to promote disulfidptosis and suppress tumor growth upon glucose limitation (Wang J. et al., 2025).

From an immunological perspective, the timing of disulfidptosis induction relative to ICB administration is critical. As an illustrative hypothesis, we propose a “prime-boost” framework: administering disulfidptosis inducers 24–48 h prior to PD-1/PD-L1 blockade might allow sufficient time for DAMP release, DC migration, and T cell priming before checkpoint blockade is applied. However, we emphasize that the optimal temporal window has not been systematically explored in disulfidptosis models and requires careful characterization. The 24–48-h interval is a working hypothesis based on general principles of DC activation kinetics, not on direct experimental evidence specific to disulfidptosis.

Beyond nanomedicine, dietary metabolic interventions—notably ketogenic diets—have shown promise in sensitizing hepatocellular carcinoma cells to disulfidptosis under glucose-starved conditions, offering a low-toxicity adjuvant strategy (Zhang et al., 2025). Additionally, small-molecule compounds including lomerizine and clioquinol as sensitizers, and nicotinamide riboside chloride (NR-CL) as a desensitizer, provide pharmacological tools for modulating disulfidptosis sensitivity, though their specificity and *in vivo* efficacy require further optimization (Chen R. et al., 2025; Zhang et al., 2025).

A critical translational consideration that warrants explicit emphasis is the potential off-target impact of disulfidptosis-inducing agents on non-malignant cells. While tumor-selective nanodelivery strategies aim to minimize systemic exposure (Chen Y. et al., 2025; Xu Y. et al., 2025; Jin et al., 2025; Yang et al., 2025), CD8^+^ T cells, dendritic cells, and intestinal epithelial cells all share metabolic vulnerabilities that may render them susceptible to disulfide stress under glucose-limited conditions. For instance, CD8^+^ T cells within the same glucose-deprived tumor microenvironment can undergo SLC7A11-dependent disulfidptosis, contributing to T cell exhaustion and immune evasion (Li and Gan, 2025; Wan et al., 2025); intestinal epithelium, which depends on robust redox homeostasis, may be particularly vulnerable to GPX4 inhibition-related toxicity (Qian et al., 2026). Thus, the therapeutic window is likely to be narrow and highly dependent on cell-type-specific SLC7A11 expression levels, NADPH compartmentalization, and the duration of drug exposure. These parameters remain largely uncharacterized and should be systematically evaluated in preclinical safety studies prior to clinical translation (Li and Gan, 2025; Qian et al., 2024).

### 5.2 Emergent resistance mechanisms: metabolic and genetic adaptation

Any proposed rheostat model must account for the tumor’s capacity to escape the setpoint. We hypothesize, as a testable prediction, that chronic or repeated disulfidptosis pressure may select for at least two classes of resistance. First, metabolic rewiring: tumors may upregulate mitochondrial NADPH production (via IDH2 or malic enzyme) to compensate for cytosolic NADPH depletion, or they may downregulate SLC7A11 surface expression to limit cystine influx—though the latter would simultaneously increase oxidative stress sensitivity. Second, immune-editing resistance: tumors that survive sub-lethal disulfide stress may acquire a more immunoevasive phenotype, upregulating CD73 to accelerate ATP degradation or secreting IL-10/TGFβ to dampen DC maturation (O'Donnell et al., 2017). These possibilities are speculative but are explicitly posed here as testable predictions for longitudinal studies in murine relapse models.

### 5.3 Current translational bottlenecks and urgent experimental gaps

Despite encouraging preclinical data, several critical hurdles impede clinical translation. Optimal sequencing and dosing remain largely unexplored. Predictive biomarkers lack standardized cut-offs (e.g., SLC7A11 H-score >150), and intratumoral heterogeneity complicates validation. The immunogenicity threshold (DAMP kinetics, ATP/adenosine ratio) is undefined, and the gut microbiota–disulfidptosis link remains speculative.

To move beyond correlation toward causal understanding, we distill the four priority experimental areas into Box 1.

Box 1 **Outstanding Questions and Priority Experiments to Validate or Falsify the Rheostat Hypothesis.**

**T cell-intrinsic causality of exhaustion**: Does T-cell-specific Slc7a11 ablation (*Slc7a11* ^fl/fl^ crossed with Cd4-Cre or Cd8-Cre) rescue TCR signaling strength (pZAP70, pERK) and cytolytic function in orthotopic murine GI cancer models? Does this overcome ICB resistance independently of tumor-cell effects? Parallel measurements of pZAP70/pERK in human tumor-infiltrating lymphocytes are required to link SLC7A11 expression to functional impairment.

**Immunogenicity threshold and DC function**: How does disulfidptosis compare side-by-side with canonical ICD inducers (e.g., oxaliplatin) regarding ATP release kinetics (0–60 min), calreticulin exposure (2–6 h), DC cross-presentation efficiency (OT-I T cell proliferation), and the ATP/adenosine ratio in the tumor bed? Does NADPH depletion impair cDC1 survival or cross-presentation capacity, and can supplementation with NADPH precursors (e.g., nicotinamide riboside) restore function? Recent evidence from RRx-001 studies (Huang et al., 2026) provides a benchmark for DAMP release kinetics and ICD induction in hepatocellular carcinoma, which can serve as a reference for future comparative studies.

**DPP7–GPX4 axis as a therapeutic node**: Does pharmacological DPP7 inhibition (e.g., Val-boroPro) or GPX4 degradation (using proteolysis-targeting chimeras [PROTACs]) enhance NK killing, DAMP release, and synergy with anti-PD-1 in orthotopic GI models? What are the on-target/off-tumor toxicities, especially to intestinal epithelium?

**Spatial and human pathological validation**: Do FLNA aggregates and intracellular disulfide content (via biotin-switch assay) in human GI tumor biopsies correlate with pZAP70^+^ CD8^+^ T cells and ICB response? How spatially heterogeneous are these markers relative to immune infiltrates and DAMP distribution across different tumor regions, as mapped by multiplex imaging (e.g., CODEX or MIBI) (Lewis et al., 2021)?

## 6 Conclusion: tuning the rheostat for immunological benefit

We conclude that disulfidptosis may function not merely as a tumor cell death pathway, but as an immunometabolic rheostat that integrates nutrient availability, redox balance, and immune cell fate into a unified decision node. Three principles emerge from this framework.

For CD8^+^ T cells, chronic sub-lethal disulfide stress is associated with progressive dysfunction—not necessarily through direct cytotoxicity, but potentially by eroding TCR signaling via F-actin destabilization, disrupting cytolytic granule polarization, and correlating with TOX-associated transcriptional changes. However, the causal chain from disulfide stress to irreversible exhaustion remains to be firmly established.

For DCs and the adaptive response, the immunogenicity of disulfidptosis is likely defined by kinetic and redox thresholds—rapid synchronous lysis that releases fully reduced HMGB1 and ATP above degradation rates may prime cross-presentation, whereas protracted or redox-compromised DAMP release may be tolerogenic or inconsequential. The field must move from asking “Is disulfidptosis immunogenic?” to defining the specific conditions under which it becomes so (Li and Gan, 2025; Galluzzi et al., 2024; Galluzzi et al., 2020; Li C. et al., 2026). The recent demonstration that RRx-001 triggers disulfidptosis coupled with DAMP-mediated ICD in hepatocellular carcinoma (Huang et al., 2026) provides a critical proof-of-concept that pharmacological induction of disulfidptosis can indeed elicit productive antitumor immunity.

The DPP7–GPX4 axis positions disulfidptosis within the innate immune checkpoint paradigm—by suppressing this death pathway, tumors evade NK surveillance and may prevent the DAMP–DC–T cell cascade, presenting a therapeutically tractable node upstream of PD-1/PD-L1 (Li R. et al., 2025). This concept awaits rigorous *in vivo* validation across multiple models.

Clinically, this framework demands context-guided strategies: DRG signatures for patient stratification, timed nanodelivery to synchronize tumor lysis with DC activation, and selective DPP7/GPX4 disruption to relieve innate suppression, all while monitoring T cell functional readouts (pZAP70, T-bet/Eomes ratio) as pharmacodynamic endpoints.

### 6.1 Falsifiability of the rheostat hypothesis

The conceptual framework we advance is intended to generate testable predictions. It would be substantially weakened by any of the following findings: T-cell-specific Slc7a11 ablation fails to rescue TCR signaling or effector function in orthotopic GI models; LDHB inhibition does not restore NADPH levels or prevent exhaustion-associated gene expression in human tumor-infiltrating lymphocytes; disulfidptotic tumor cells fail to elicit productive DC cross-presentation even under optimized conditions of synchronous lysis and ectonucleotidase blockade; or DPP7–GPX4 disruption does not enhance NK-mediated killing or synergize with anti-PD-1 *in vivo*. Conversely, positive results in these experiments would strengthen the model and support its clinical translation. We offer this framework not as a closed doctrine, but as a roadmap for experimental interrogation. By shifting the conceptual lens from “targeting a death pathway” to “tuning an immunometabolic rheostat,” the field can subvert the metabolically enforced immune privilege of GI malignancies and expand the benefits of immunotherapy to patients who currently derive little or no response (Chen Y. et al., 2025; Xu S. et al., 2025).

## References

[B1] Chen Y. Zhang D. Yang H. Wu J. He W. (2025). Advances in the study of disulfidptosis in digestive tract tumors. Discov. Oncol. 16 (1), 186. 10.1007/s12672-025-01875-y 39954025 PMC11829889

[B2] Chen R. You J. Weng S. Zhao T. (2025). Disulfidptosis mechanisms and therapeutic implications in cancer metabolic reprogramming and future perspectives. Discov. Oncology 16 (1), 1814. 10.1007/s12672-025-03538-4 41042274 PMC12495009

[B3] Galluzzi L. Vitale I. Warren S. Adjemian S. Agostinis P. Martinez A. B. (2020). Consensus guidelines for the definition, detection and interpretation of immunogenic cell death. J. Immunotherapy Cancer 8 (1). 10.1136/jitc-2019-000337 32209603 PMC7064135

[B4] Galluzzi L. Guilbaud E. Schmidt D. Kroemer G. Marincola F. M. (2024). Targeting immunogenic cell stress and death for cancer therapy. Nat. Rev. Drug Discovery 23 (6), 445–460. 10.1038/s41573-024-00920-9 38622310 PMC11153000

[B5] Godfrey D. I. Le Nours J. Andrews D. M. Uldrich A. P. Rossjohn J. (2018). Unconventional T cell targets for cancer immunotherapy. Immunity 48 (3), 453–473. 10.1016/j.immuni.2018.03.009 29562195

[B6] Huang H. He Y. Chen J. Liao Y. Mo S. Qin W. (2026). RRx-001 inhibits G6PD to deplete NADPH and trigger disulfidptosis coupled with DAMP-Mediated immunogenic cell death in hepatocellular carcinoma. Cell Death Discovery 12 (1), 194. 10.1038/s41420-026-03032-y 41888099 PMC13144330

[B7] Jiang W. Yang H. Li R. Li Y. Xia S. (2026). Disulfidptosis-related genes signature predicts prognosis and immune microenvironment in Colon cancer. Front. Molecular Biosciences 13, 1756041. 10.3389/fmolb.2026.1756041 41684457 PMC12890678

[B8] Jin X. K. Zhang S. K. Zhang S. M. Liang J. L. Yan X. Lin Y. T. (2025). Disrupting intracellular homeostasis by copper-based nanoinducer with multiple enzyme-mimicking activities to induce Disulfidptosis-Enhanced pyroptosis for tumor immunotherapy. Adv. Materials Deerf. Beach, Fla 37 (1), e2410957. 10.1002/adma.202410957 39468892

[B9] Josefowicz S. Z. Lu L. F. Rudensky A. Y. (2012). Regulatory T cells: mechanisms of differentiation and function. Annu. Review Immunology 30, 531–564. 10.1146/annurev.immunol.25.022106.141623 22224781 PMC6066374

[B10] Le D. T. Durham J. N. Smith K. N. Wang H. Bartlett B. R. Aulakh L. K. (2017). Mismatch repair deficiency predicts response of solid tumors to PD-1 blockade. Sci. New York, NY 357 (6349), 409–413. 10.1126/science.aan6733 28596308 PMC5576142

[B11] Lewis S. M. Asselin-Labat M. L. Nguyen Q. Berthelet J. Tan X. Wimmer V. C. (2021). Spatial omics and multiplexed imaging to explore cancer biology. Nat. Methods 18 (9), 997–1012. 10.1038/s41592-021-01203-6 34341583

[B12] Li Q. Gan B. (2025). Disulfidptosis meets antitumour immunity. Nat. Cell Biology 27 (6), 886–887. 10.1038/s41556-025-01679-w 40473855

[B13] Li T. Song Y. Wei L. Song X. Duan R. (2024). Disulfidptosis: a novel cell death modality induced by actin cytoskeleton collapse and a promising target for cancer therapeutics. Cell Communication Signaling CCS 22 (1), 491. 10.1186/s12964-024-01871-9 39394612 PMC11470700

[B14] Li R. Wang X. Liu J. Cai Z. Li Z. Tao Q. (2025). DPP7 promotes colorectal cancer progression through GPX4-Dependent suppression of disulfidptosis and immune evasion. J. Cellular Mol. Med. 29 (12), e70660. 10.1111/jcmm.70660 40576169 PMC12203401

[B15] Li Q. Huang R. Lv L. Ying H. Wu Y. Huang Y. (2025). FLNA, a disulfidptosis-related gene, modulates tumor immunity and progression in colorectal cancer. Cell. and Molecular Biology Letters 30 (1), 92. 10.1186/s11658-025-00761-3 40713509 PMC12297336

[B16] Li C. Zhu L. Wang Y. Zhao L. Lin X. Sun Z. (2026). Spatiotemporal control of immunogenic cell death: rewiring tumor-immune dialogues for next-generation immunotherapy. Front. Immunol. 17, 1784935. 10.3389/fimmu.2026.1784935 41853278 PMC12992043

[B17] Li W. Deng J. Zhang H. Zhang M. Kang C. Yu Z. (2026). The role of disulfidptosis-driven tumor microenvironment remodeling in pancreatic cancer progression. Front. Immunol. 17, 1747560. 10.3389/fimmu.2026.1747560 41884834 PMC13008902

[B18] Liu X. Nie L. Zhang Y. Yan Y. Wang C. Colic M. (2023). Actin cytoskeleton vulnerability to disulfide stress mediates disulfidptosis. Nat. Cell Biol. 25 (3), 404–414. 10.1038/s41556-023-01091-2 36747082 PMC10027392

[B19] Liu X. Zhuang L. Gan B. (2024). Disulfidptosis: disulfide stress-induced cell death. Trends Cell Biology 34 (4), 327–337. 10.1016/j.tcb.2023.07.009 37574347

[B20] Liu X. Ding Q. Zhang H. Zhang X. Chen Q. Weng S. (2025). The CD39-CD73-adenosine axis: master regulator of immune evasion and therapeutic target in pancreatic ductal adenocarcinoma. Biochimica biophysica acta Rev. cancer 1880 (5), 189443. 10.1016/j.bbcan.2025.189443 40930263

[B21] Llovet J. M. Castet F. Heikenwalder M. Maini M. K. Mazzaferro V. Pinato D. J. (2022). Immunotherapies for hepatocellular carcinoma. Nat. Reviews Clin. Oncology 19 (3), 151–172. 10.1038/s41571-021-00573-2 34764464

[B22] O'Donnell J. S. Long G. V. Scolyer R. A. Teng M. W. Smyth M. J. (2017). Resistance to PD1/PDL1 checkpoint inhibition. Cancer Treatment Reviews 52, 71–81. 10.1016/j.ctrv.2016.11.007 27951441

[B23] Qian S. Chen G. Li R. Ma Y. Pan L. Wang X. (2024). Disulfide stress and its role in cardiovascular diseases. Redox Biol. 75, 103297. 10.1016/j.redox.2024.103297 39127015 PMC11364009

[B24] Qian S. Pan L. Chen G. Qiu F. Ma Y. Li R. (2026). Disulfidptosis: a novel cell death mechanism with pathological significance and therapeutic potential in diseases. Pharmacol. Rev. 78, 100127. 10.1016/j.pharmr.2026.100127 41818911

[B25] Qiu H. Liu J. Shao N. Zhao J. Chen C. Jiang Y. (2025). SLC7A11 as a bridge between ferroptosis and disulfidptosis: a promising target for tumor treatment. Cell Communication Signaling CCS 23 (1), 460. 10.1186/s12964-025-02447-x 41146208 PMC12557889

[B26] Senft D. (2025). Exhausting bonding. Nat. Reviews Cancer 25 (8), 571. 10.1038/s41568-025-00849-0 40603726

[B27] Shi J. Zhao L. Wang K. Lin J. Shen J. (2025). Disulfidptosis classification of pancreatic carcinoma reveals correlation with clinical prognosis and immune profile. Hereditas 162 (1), 26. 10.1186/s41065-025-00381-z 39987145 PMC11846472

[B28] Wan J. Shi J. H. Shi M. Huang H. Zhang Z. Li W. (2025). Lactate dehydrogenase B facilitates disulfidptosis and exhaustion of tumour-infiltrating CD8(+) T cells. Nat. Cell Biol. 27 (6), 972–982. 10.1038/s41556-025-01673-2 40461882

[B29] Wang Y. Guo F. Song W. Liu Y. Xiang Q. (2025). Integrated genomic analysis identifies clinically relevant molecular subtypes and Disulfidptosis-Related prognostic signature of gastric cancer. J. Immunotherapy Hagerst. Md 1997 48 (9), 343–357. 10.1097/cji.0000000000000570 40653647

[B30] Wang J. Chen J. Fan K. Wang M. Gao M. Ren Y. (2025). Inhibition of endoplasmic reticulum stress cooperates with SLC7A11 to promote disulfidptosis and suppress tumor growth upon glucose limitation. Adv. Sci. Weinheim, Baden-Wurttemberg, Ger. 12 (7), e2408789. 10.1002/advs.202408789 39739602 PMC11831432

[B31] Wherry E. J. Kurachi M. (2015). Molecular and cellular insights into T cell exhaustion. Nat. Rev. Immunol. 15 (8), 486–499. 10.1038/nri3862 26205583 PMC4889009

[B32] Xiao F. Li H. L. Yang B. Che H. Xu F. Li G. (2024). Disulfidptosis: a new type of cell death. Apoptosis. An Inter. J. Programmed Cell Death 29 (9-10), 1309–1329. 10.1007/s10495-024-01989-8 38886311 PMC11416406

[B33] Xu Y. Ming X. Qi J. Huang Z. Zhu H. Wu M. (2025). Disulfidptosis nanoinducer interrupts tumor metabolic privilege to boost sustained immunotherapy. ACS Nano 19 (33), 30303–30321. 10.1021/acsnano.5c08432 40788631

[B34] Xu S. Chen Z. Chen X. Chu H. Huang X. Chen C. (2025). Interplay of disulfidptosis and the tumor microenvironment across cancers: implications for prognosis and therapeutic responses. BMC Cancer 25 (1), 1113. 10.1186/s12885-025-14246-1 40597807 PMC12210770

[B35] Xu Y. Kong X. Li J. Xiang D. (2026). “Disulfidptosis-related signatures identify SPARC(+)/CYR61(+) stromal cells mediating immunosuppression in gastric cancer,”Cell. Oncol. Dordrecht, Netherlands. 10.1007/s13402-026-01231-4 PMC13562495 42360660

[B36] Yang Y. Zhang S. Hu X. Hu C. Lv Y. Pei Y. (2025). Oxygen-driven Prussian blue nanomotor promotes cancer immunotherapy by disrupting redox state-induced tumor disulfide death. J. Colloid Interface Sci. 700 (Pt 2), 138404. 10.1016/j.jcis.2025.138404 40680542

[B37] Yin J. Li J. Wang H. (2025). Disulfidptosis: a novel gene-based signature predicts prognosis and immunotherapy efficacy of pancreatic adenocarcinoma. Discov. Oncology 16 (1), 308. 10.1007/s12672-025-02053-w 40072658 PMC11904034

[B38] Yin Y. Sun Y. Yao H. Yu F. Jia Q. Hu C. (2025). TMEM105 modulates disulfidptosis and tumor growth in pancreatic cancer via the β-catenin-c-MYC-GLUT1 axis. Int. J. Biol. Sci. 21 (5), 1932–1948. 10.7150/ijbs.104598 40083702 PMC11900826

[B39] Yu J. Yin L. Li M. Li Q. Qin X. Qin L. (2025). Disulfidptosis-related LncRNA signatures in gastric cancer: regulation of MYH10-driven cytoskeletal remodeling and therapeutic implications. Discov. Oncol. 16 (1), 1385. 10.1007/s12672-025-03160-4 40694286 PMC12394114

[B40] Zhang Y. Guo Y. He W. Zhao Y. Feng Z. Shi M. (2025). Deciphering the rules of disulfidptosis: a genome-wide signature for identifying disulfidptosis-related genes and analyzing hepatocellular carcinoma chemotherapy sensitivities. Free Radical Biol. Med. 241, 914–932. 10.1016/j.freeradbiomed.2025.10.278 41138962

